# A mitochondrial ferroptosis-related gene signature predicts prognosis and immune landscape in colon cancer

**DOI:** 10.3389/fmed.2025.1614012

**Published:** 2025-09-01

**Authors:** Hou Wang

**Affiliations:** ^1^Department of Endocrinology and Metabolism, Ningbo No. 2 Hospital, Ningbo, Zhejiang, China; ^2^Department of Endocrine and Metabolic Diseases, Shanghai Institute of Endocrine and Metabolic Diseases, Ruijin Hospital, Shanghai Jiao Tong University School of Medicine, Shanghai, China; ^3^Key Laboratory for Endocrine and Metabolic Diseases of the National Health Commission of the PR China, Shanghai National Clinical Research Center for Metabolic Diseases, Shanghai National Center for Translational Medicine, Ruijin Hospital, Shanghai Jiao Tong University School of Medicine, Shanghai, China

**Keywords:** colon cancer, prognostic biomarker, mitochondrial ferroptosis, tumor microenvironment, immunotherapy, drug sensitivity

## Abstract

**Background:**

Colon cancer is a highly aggressive gastrointestinal malignancy with significant global health implications. Although mitochondrial ferroptosis-related genes have been implicated in colon cancer progression, their prognostic significance remains inadequately understood.

**Methods:**

We conducted a comprehensive analysis of the expression patterns and prognostic value of mitochondrial ferroptosis-related genes in patients with colon cancer, utilizing data from the TCGA and GEO databases. A prognostic risk model was established, followed by evaluations of the tumor microenvironment (TME), immune cell infiltration, tumor mutation burden (TMB), and predicted drug sensitivity. P4HA1, recognized as an important mitochondrial ferroptosis-associated gene, was selected for functional characterization using *in vitro* experiments.

**Results:**

Four key mitochondrial ferroptosis-associated genes—PDSS2, GRSF1, SLC39A8, and P4HA1—were identified. A nomogram combining the risk score and pTNM stage was constructed to predict patient outcomes. Immune microenvironment analysis revealed distinct differences in immune cell infiltration between the high- and low-risk groups. The risk score was significantly correlated with the expression of TME-related genes and immune checkpoint molecules, suggesting a more immunosuppressive microenvironment in high-risk patients. Furthermore, integrating the risk score with TMB enhanced the accuracy of survival prediction. Silencing P4HA1 markedly reduces the proliferative and migratory abilities of colorectal cancer cells *in vitro*.

**Conclusion:**

This mitochondrial ferroptosis-based risk model represents a promising prognostic biomarker and may offer valuable insights for personalized treatment strategies in colon cancer management. P4HA1 facilitates the advancement of colorectal cancer, while its suppression diminishes the *in vitro* proliferation and migration of colorectal cancer cells.

## Introduction

Colorectal cancer (CRC) is the third most commonly diagnosed malignancy and the second leading cause of cancer-related mortality worldwide, with approximately 1.9 million new cases and 900,000 deaths reported in 2020 (1). Although CRC incidence remains highest in highly developed countries, a rising number of cases are now being observed in middle- and low-income regions, largely due to the increasing adoption of Westernized lifestyles. Moreover, there is a concerning upward trend in the incidence of early-onset CRC, particularly among younger populations (2, 3). For patients with metastatic colorectal cancer (mCRC), survival statistics remain suboptimal: approximately 70–75% survive beyond 1 year, 30–35% live beyond 3 years, and fewer than 20% reach 5-year survival post-diagnosis (4). In cases where surgical resection is not an option, systemic therapies—including cytotoxic chemotherapy, targeted agents such as anti-growth factor receptor antibodies, immunotherapy, or their combinations—remain the cornerstone of treatment (5). These concerning epidemiological trends highlight the urgent need for more reliable prognostic biomarkers and novel molecular targets to enhance therapeutic strategies in colorectal cancer.

Mitochondrial function is closely linked to the regulation of ferroptosis. Accumulating evidence indicates that mitochondrial impairment and dysfunction exacerbate oxidative stress, thereby promoting ferroptotic cell death (6, 7). As the primary sites for intracellular ATP production, mitochondria also serve as the main source of reactive oxygen species (ROS). Excessive mitochondrial ROS can initiate ferroptosis by driving lipid peroxidation (8). Additionally, components of the mitochondrial electron transport chain and proton-pumping mechanisms play critical roles in the induction of ferroptosis. Under conditions of ATP depletion, AMP-activated protein kinase (AMPK) is activated, leading to the inhibition of fatty acid synthesis, which subsequently suppresses ferroptotic activity (9, 10). Beyond energy production, mitochondria participate in biosynthetic and metabolic pathways through the tricarboxylic acid (TCA) cycle and associated metabolic fluxes, which may further modulate ferroptosis by influencing electron transport and lipid metabolism (7, 11).

Beyond its role in inducing tumor cell death, ferroptosis also contributes to immunosuppression within the tumor microenvironment (TME) by affecting both innate and adaptive immune responses (12). Emerging evidence suggests that innate immune cells in the TME, such as tumor-associated macrophages (TAMs) and tumor-associated neutrophils (TANs), play critical roles in regulating iron metabolism by modulating iron availability to cancer cells (13). Macrophages, in particular, recycle iron from senescent red blood cells and reintroduce it into circulation, thereby supplying essential iron for various metabolic processes (14). In this context, cancer cells function as iron consumers, whereas macrophages and neutrophils act as iron providers.

Within the TME, TAMs promote cancer cell proliferation, angiogenesis, and contribute to immunosuppression and therapeutic resistance (15). Although direct evidence of Fe^2+^ secretion by TAMs and TANs remains limited, elevated local Fe^2+^ concentrations may contribute to ferroptosis in both cancer cells and infiltrating T cells. Moreover, TAMs and TANs may impair the maturation of antigen-presenting cells (APCs) and suppress effective antigen presentation, further dampening anti-tumor immune responses (16). Similarly, neutrophils produce iron-binding proteins such as lipocalin-2 (Lcn2) and lactoferrin, which influence iron homeostasis in the TME (17). Collectively, these findings suggest that modulating Fe^2+^ levels within the TME could represent a promising strategy to enhance the efficacy of existing cancer therapies.

Given the pivotal role of mitochondrial ferroptosis in tumor progression, identifying biomarkers associated with this process represents a promising avenue for improving prognostic prediction in colorectal cancer (CRC). Although mitochondrial ferroptosis is increasingly recognized as a key mechanism in cancer biology, its underlying molecular pathways and therapeutic potential remain inadequately understood. While several prognostic models have been proposed for CRC survival prediction (18–21), few have specifically focused on ferroptosis-related signatures capable of predicting both prognosis and immunotherapy response in colon cancer.

In this study, we constructed a mitochondrial ferroptosis-based risk scoring model and systematically investigated its associations with tumor microenvironment (TME) features, including immune cell infiltration, immune checkpoint expression, and predicted immunotherapy response. Additionally, we evaluated drug sensitivity across 198 compounds to identify potential therapeutic agents. Overall, our risk model not only serves as a robust prognostic biomarker for colon cancer but also provides novel insights into personalized treatment strategies. By linking mitochondrial ferroptosis to an immunosuppressive TME, this model contributes to a deeper understanding of colon cancer pathogenesis and offers a foundation for the development of improved therapeutic approaches.

## Materials and methods

### Data collection

This research utilized publicly accessible datasets for genomic and clinical data. RNA-seq data from 455 colon adenocarcinoma (COAD) samples, along with their corresponding clinical information, were sourced from The Cancer Genome Atlas (TCGA) through UCSC Xena. For external validation of our prognostic gene signature, we utilized the GEO dataset GSE39582, which includes data from 585 samples. In our study, we initially retrieved 2,091 ferroptosis-related genes from the FerrDb database and 2,030 mitochondrial genes from the MitoCarta3.0 database. Ferroptosis-related genes were obtained from the FerrDb database^1^ (22), and mitochondrial genes were compiled from the MitoCarta3.0 database^2^ (23). To enhance the reliability of gene selection, we additionally reviewed relevant literature to confirm their functional involvement in ferroptosis or mitochondrial activity in the context of cancer (23–26). By intersecting the two datasets, a total of 151 overlapping genes were identified, all of which are implicated in both ferroptosis and mitochondrial function. Additional validation of these genes was carried out using expression data from cancer cohorts, complemented by functional annotation and pathway enrichment analysis to establish their role in mitochondrial ferroptosis.

### Construction and evaluation of a mitochondrial ferroptosis-related prognostic model

Differential gene expression analysis was performed using the “limma” R package to identify differentially expressed genes (DEGs) between tumor and normal samples, as well as between high- and low-risk groups (criteria: |log2 FC| > 1.3, adjusted *p* < 0.05). Univariate Cox regression was applied to select genes significantly associated with overall survival (OS), followed by LASSO regression to refine the prognostic signature. A 4-gene risk score was constructed as follows:


$$\text{Risk}\,\text{score}=\Sigma \,{\text{expgenei}}^{*}\,\beta \,i$$

where Expi is the expression level of gene i, and βi is the corresponding LASSO-derived coefficient. Based on the median score, patients were stratified into high- and low-risk groups. Clinical variables, such as age, gender, and TNM stage, were collected from TCGA. Both univariate and multivariate Cox regression analyses were performed to assess the independent prognostic value of the model (*p* < 0.05). Validation of the model was conducted using the GSE39582 cohort, incorporating time-dependent ROC curves, Kaplan-Meier survival analysis, and Harrell’s C-index. Gene annotations were validated via the NCBI database.

### Nomogram construction and validation

To construct the prognostic nomogram, we integrated survival time, survival status, and three selected features using the R package “rms.” Candidate variables were first identified through univariate Cox regression analysis (*p* < 0.05), followed by stepwise multivariate Cox regression using the Akaike Information Criterion (AIC) to determine the most informative features. The final nomogram was developed based on the selected variables and assessed in colon cancer samples. A nomogram was developed by combining the risk score with clinical variables using Cox regression results, aiming to predict 3-, 4-, and 5-year survival probabilities. The predictive performance of the nomogram was assessed using calibration plots, receiver operating characteristic (ROC) curves, and the concordance index (C-index). Calibration was conducted via bootstrap resampling with 1,000 iterations to compare predicted and observed survival probabilities at 3-, 4-, and 5-year time points.

### Protein expression validation using immunohistochemistry from the human protein atlas

To validate the protein expression levels of the prognostic genes identified in the model, immunohistochemical (IHC) staining data from the Human Protein Atlas (HPA) database were utilized. The HPA provides extensive IHC staining data for various human tissues. IHC images for the four core genes were retrieved from the HPA database to assess their expression patterns in both tumor and adjacent normal tissues. Representative IHC images for each gene (GRSF1, P4HA1, PDSS2, and SLC39A8) are shown in the corresponding figures. These images were used to confirm the protein-level expression of the candidate genes and evaluate their potential as biomarkers in colon cancer.

### Gene ontology, kyoto encyclopedia of genes and genomes analyses

Gene Ontology (GO) analysis, KEGG pathway enrichment, and Gene Set Enrichment Analysis (GSEA) were conducted to identify the biological functions and pathways associated with DEGs. These analyses were performed in R using the “clusterProfiler,” “org.Hs.eg.db,” “enrichplot,” and “ggplot2” packages, with significance determined at an FDR < 0.05.

### Tumor microenvironment analysis

Stromal and immune scores were calculated using the ESTIMATE algorithm. TME-related biomarkers were sourced from the MSigDB.^3^ Immune cell composition was analyzed using the CIBERSORT algorithm via the “immunedeconv” R package. The Tumor Immunophenotype (TIP) platform, which integrates ssGSEA (single-sample Gene Set Enrichment Analysis) and CIBERSORT, was used to assess immune cell infiltration within tumors, providing insights into immune responses to cancer.

### Prediction of therapeutic response

Drug sensitivity for 198 anticancer compounds was predicted using the “oncoPredict” R package, with IC50 values estimated from the GDSC database. Additionally, the Tumor Immune Dysfunction and Exclusion (TIDE) algorithm was applied to predict the potential efficacy of immune checkpoint inhibitors.

### Mutation landscape analysis

Somatic mutation data from colon cancer patients were downloaded from cBioPortal. Mutation profiles and tumor mutational burden (TMB) were analyzed using the “maftools” R package, and visualized with waterfall plots.

### Ethics approval statement

This study received approval from the Human Research Ethics Committee of Ruijin Hospital (Approval No. 2020–115) and was carried out in compliance with the ethical principles outlined in the Declaration of Helsinki.

### Cell lines and culture

Human colorectal cancer (CRC) cell lines RKO and HCT116 were purchased from the American Type Culture Collection (ATCC, United States), cells were authenticated by STR profiling and free of mycoplasma contamination. Cells cultured in DMEM medium (Meilunbio, Dalian, China) containing 10% fetal bovine serum (Gibco, Grand Island, NY, United States), 100 U mL^–1^ of penicillin, and 100 μg mL^–1^ of streptomycin, in a humidity culture incubator at 37°C with 5% CO_2_.

### Lentiviral-mediated knockdown of P4HA1

For the knockdown of P4HA1, target shRNA sequences (5’–3’ATTTGAGCTATGCGGTATATC) were subcloned into pGreenPuro (CMV) vector. For shRNA lentivirus infection, target cells were seeded in a 6-well plate 24 h before infection and were grown to 60–80% confluency upon transduction. Culture medium was removed, and cells were incubated with virus supernatant along with 10 μg/mL polybrene (Sigma) overnight. Virus-containing medium was replaced with fresh medium. Puromycin (Sigma) (10 μg/mL) was applied to kill non-infected cells 48 h after infection to produce stably transfected cells (RKO/shP4HA1, HCT116/shP4HA1).

### Western blotting

Total protein was extracted from cells using RIPA lysis buffer (Kangwei, Beijing, China) supplemented with phosphatase inhibitors (Roche). Protein concentration was determined by BCA assay (Pierce, United States). Equal amounts (20 μg) of protein were resolved via SDS-PAGE and transferred to 0.22 μm PVDF membranes (Millipore, United States). After blocking with 5% non-fat milk in TBST, membranes were incubated overnight at 4°C with primary antibodies against GAPDH (Proteintech), P4HA1 (Abcam, ab127564), and PD-L1 (Abcam, ab239749), followed by HRP-conjugated secondary antibody (Thermo Fisher). Signals were visualized using ECL substrate (Thermo, United States) and imaged with the Tanon 5200 system (Tanon, China).

### CCK8 assays

Cell proliferation was assessed using the Cell Counting Kit-8 (CCK-8; Dojindo, Japan) following the manufacturer’s instructions. Briefly, cells were seeded into 96-well plates at a density of 2 × 10^3^ cells/well in 200 μL of complete medium. At the indicated time points, the culture medium was removed and replaced with 100 μL of fresh medium containing 10% CCK-8 solution. The cells were incubated at 37°C for 2 h, and the absorbance was measured at 450 nm using a microplate reader (BioTek, United States). The optical density (OD450) value on day 1 was used as a baseline for normalization, and relative proliferation was calculated by comparing OD values from subsequent days to this baseline. All experiments were performed in triplicate.

### Migration and invasion assays

Cell migration and invasion were evaluated using Transwell chambers (8 μm pore, Corning, United States). For migration, 1 × 10^5^ cells in serum-free medium were seeded in the upper chamber; medium with 10% FBS was added to the lower chamber. After 12 h, migrated cells on the lower surface were fixed and stained with 0.1% crystal violet. For invasion, the upper chambers were precoated with diluted Matrigel (BD Biosciences, United States), and cells were incubated for 24 h before staining. Cells in five random fields were counted under a microscope, and the average was recorded.

### Wound-healing assays

For wound-healing assays, 4 × 10^5^ cells/well were seeded onto 24-well plates and cell growth was recorded at 0 h and 24 h. The cell migration rate was analyzed using the Image J software.

### Evaluation of malondialdehyde, 4-hydroxynonenal and iron level

To evaluate the ferroptosis level in 9 CRC samples (collected in the Ruijin Hospital), the MDA, 4-HNE and iron levels were detected. The MDA concentration, 4-HNE concentration and iron concentration in cell lysates were assessed using the Lipid Peroxidation (MDA) Assay Kit (Sigma-Aldrich, Cat #: MAK085), Lipid Peroxidation (4-HNE) Assay Kit (Abcam, Cat #: ab238538) and Iron Assay Kit (Sigma-Aldrich, Cat #: MAK025) according to the manufacturer’s instructions.

### Statistical analysis

All statistical analyses were conducted using R (v4.3.3) and GraphPad Prism (v10.0.1). Statistical tests included Student’s *t*-test, chi-square test, Spearman correlation, and Cox proportional hazards regression. Predictive accuracy was assessed using the concordance index (C-index). A *p*-value < 0.05 was considered statistically significant.

## Results

### Identification of mitochondrial ferroptosis-associated DEGs and functional annotation in COAD

**FIGURE 1:**
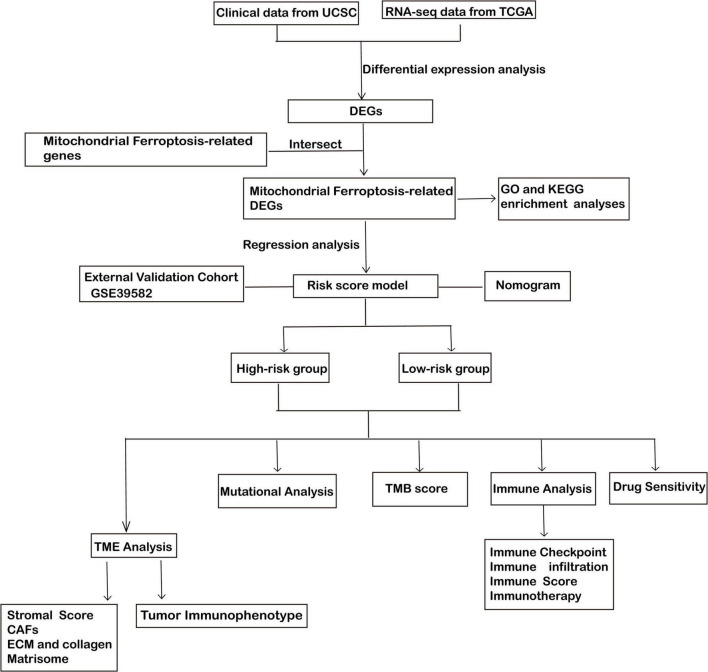
Workflow diagram: a schematic representation of the study design and the corresponding analytical steps.

**FIGURE 2:**
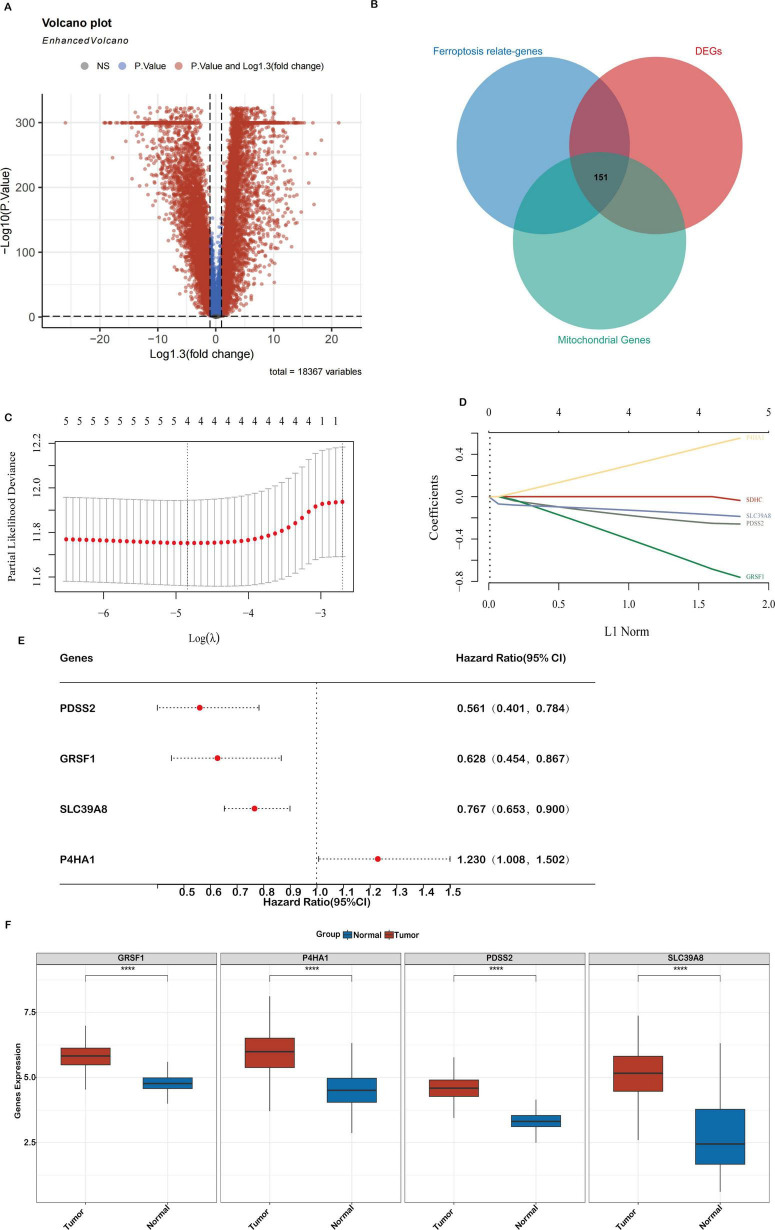
Differential gene expression (DEGs) related to mitochondrial ferroptosis and the construction of a prognostic model in the TCGA-COAD cohort **(A)**. A volcano plot showing the expression differences between COAD tumor and normal tissues, highlighting 10,884 genes. **(B)** A Venn diagram depicting the overlap between DEGs, ferroptosis-related genes, and mitochondrial genes, resulting in 151 key genes. **(C,D)** LASSO regression analyses of the 4 genes associated with overall survival (OS), including cross-validation to select the optimal tuning parameter (log[λ] on the x-axis and partial likelihood deviance on the y-axis, with red dots indicating deviations ± standard error). **(E)** A forest plot illustrating the prognostic significance of 4 genes, with their impact on patient outcomes. **(F)** Expression levels of the 4 core genes in the TCGA-COAD cohort. Statistical significance is marked as ***P < 0.001, **P < 0.01, *P < 0.05.

To elucidate genes involved in mitochondrial ferroptosis within COAD (Colon Adenocarcinoma), we conducted an integrated transcriptomic analysis. An overview of the research workflow is presented in Figure 1. A total of 10,884 differentially expressed genes (DEGs) were identified when comparing COAD tumor tissues with adjacent normal samples, comprising 5,887 upregulated and 4,997 downregulated genes, as visualized in the volcano plot (Figure 2A). A total of 2,091 ferroptosis-related genes were retrieved from the FerrDb database, and 2,030 mitochondrial genes were obtained from the MitoCarta3.0 database. These datasets were intersected with the DEGs, and a total of 151 mitochondrial ferroptosis-associated genes were identified (Figure 2B).

GO enrichment analysis showed that these genes were mainly involved in oxidative stress and apoptosis (Supplementary Figure S1A). They were predominantly located in mitochondrial structures, especially the membrane and envelope. Molecular functions were enriched in catalytic and protein-binding activities. KEGG analysis revealed significant enrichment in mitophagy and NOD-like receptor signaling pathways (Supplementary Figure S1B), both associated with colorectal cancer. Impaired mitophagy can lead to mitochondrial dysfunction and ROS accumulation, while abnormal activation of the NLR pathway, particularly via the NLRP3 inflammasome, promotes chronic inflammation and immune evasion, influencing ferroptosis and the tumor microenvironment.

### Development and validation of a prognostic signature based on mitochondrial ferroptosis-related genes in colon cancer

To construct a prognostic risk model based on mitochondrial ferroptosis-related differentially expressed genes (DEGs), we initially performed univariate Cox regression analysis on 151 candidate genes, identifying those significantly associated with overall survival in colon adenocarcinoma (COAD) patients. These candidate prognostic genes were subsequently subjected to feature selection using the Least Absolute Shrinkage and Selection Operator (LASSO) regression algorithm. A 10-fold cross-validation procedure was employed to avoid overfitting and determine the optimal regularization parameter. The value of λ corresponding to the minimum partial likelihood deviance was selected as the optimal penalty parameter for model construction. Ultimately, four hub genes—PDSS2, GRSF1, SLC39A8, and P4HA1—were identified as key components of the prognostic model (Figures 2C,D). The hazard ratios (HRs) for these genes are shown in Figure 2E. Finally, a prognostic risk score was established based on their expression levels and coefficients as follows: Riskscore = (-0.2466)*PDSS2 + (-0.6606)*GRSF1 + (-0.1669)*SLC39A8 + (0.4738)*P4HA1.

Analysis of the TCGA-COAD dataset confirmed distinct expression patterns of mitochondrial ferroptosis-related genes. Specifically, PDSS2, GRSF1, SLC39A8 and P4HA1 were significantly upregulated in tumor tissues compared to normal samples (Figure 2F). These alterations suggest a crucial role for these genes in shaping the tumor immune microenvironment and influencing colon cancer progression.

**FIGURE 3:**
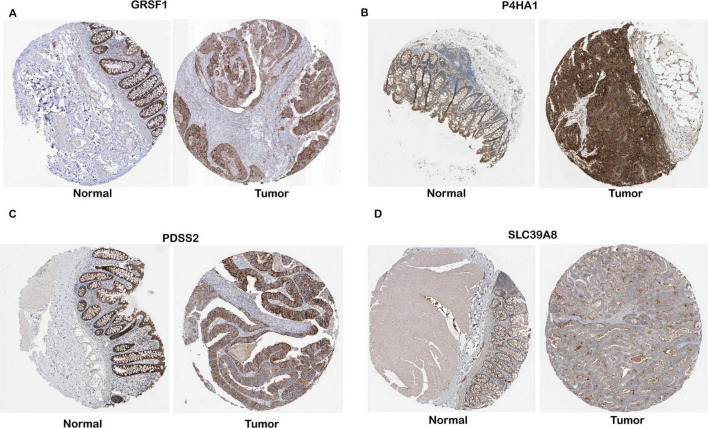
Protein-level validation of four gene expressions in COAD tissues. IHC data for GRSF1, P4HA1, PDSS2, and SLC39A8 were obtained from the Human Protein Atlas (HPA), and expression levels were interpreted based on the qualitative annotations provided by the HPA database. **(A–D)** Representative IHC images for GESF1, P4HA1, PDSS2, and SLC39A8, respectively.

Specifically, analysis of proteomic data from the Human Protein Atlas (HPA) revealed that PDSS2, GRSF1, SLC39A8, and P4HA1 are markedly upregulated in tumor tissues compared to normal samples (Figure 3). In order to verify the protein-level expression of the four key genes, we examined immunohistochemistry (IHC) data from the Human Protein Atlas (HPA). GRSF1 showed strong staining in colon cancer tissues, while no expression was detected in normal endothelial cells. P4HA1 exhibited high cytoplasmic/membranous staining intensity in tumor tissues, in contrast to weak expression in normal tissues. Similarly, PDSS2 showed strong cytoplasmic/membranous staining in tumor tissues, whereas the staining in normal tissues was low and weak. SLC39A8 was highly expressed in tumor tissues with strong intensity, while moderate expression was observed in normal tissues. All evaluations were based on qualitative annotations provided by the HPA database. Given that the HPA provides comprehensive protein expression profiles and subcellular localization information, this significant upregulation at the protein level suggests that these genes may play crucial roles in tumor development and progression by modulating key cellular processes underlying the malignant phenotype.

### Prognostic evaluation of the mitochondrial ferroptosis-based risk model in TCGA and GEO cohorts

**FIGURE 4:**
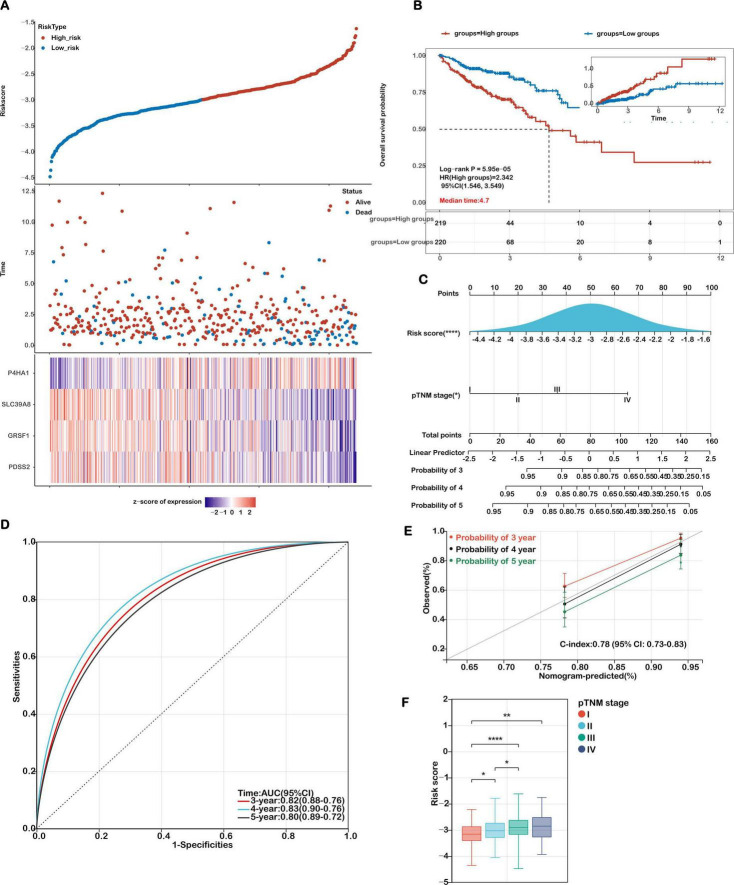
Evaluation of the prognostic model in the training cohort. **(A)** Distribution plots showing risk scores, survival status (blue for deceased, red for alive), and the expression of the 4 model genes in the TCGA-COAD training set. **(B)** Kaplan-Meier survival curves comparing overall survival between high-risk and low-risk groups. **(C)** A nomogram integrating the risk score with relevant clinical features. **(D)** Receiver Operating Characteristic (ROC) curves were generated to evaluate the predictive performance of the risk score for 3-, 4-, and 5-year overall survival. **(E)** Calibration curves illustrating the concordance between predicted and actual survival probabilities at 3, 4, and 5 years. **(F)** Analysis of the association between risk scores and TNM stage in COAD patients. Statistical significance is marked as ****P < 0.0001, **P < 0.01, *P < 0.05.

Patients from the TCGA-COAD cohort were stratified into high- and low-risk groups using the median risk score. A clear distribution of risk scores, survival outcomes, and expression levels of the four genes is shown in Figure 4A. Kaplan–Meier survival analysis demonstrated that patients in the high-risk group had significantly shorter OS than those in the low-risk group (*P* = 5.95e-05; Figure 4B). Receiver Operating Characteristic (ROC) curve analysis showed good predictive performance for the 3-, 4-, and 5-year OS, with area under the curve (AUC) values of 0.71, 0.75, and 0.74, respectively (Supplementary Figure S2).

**FIGURE 5:**
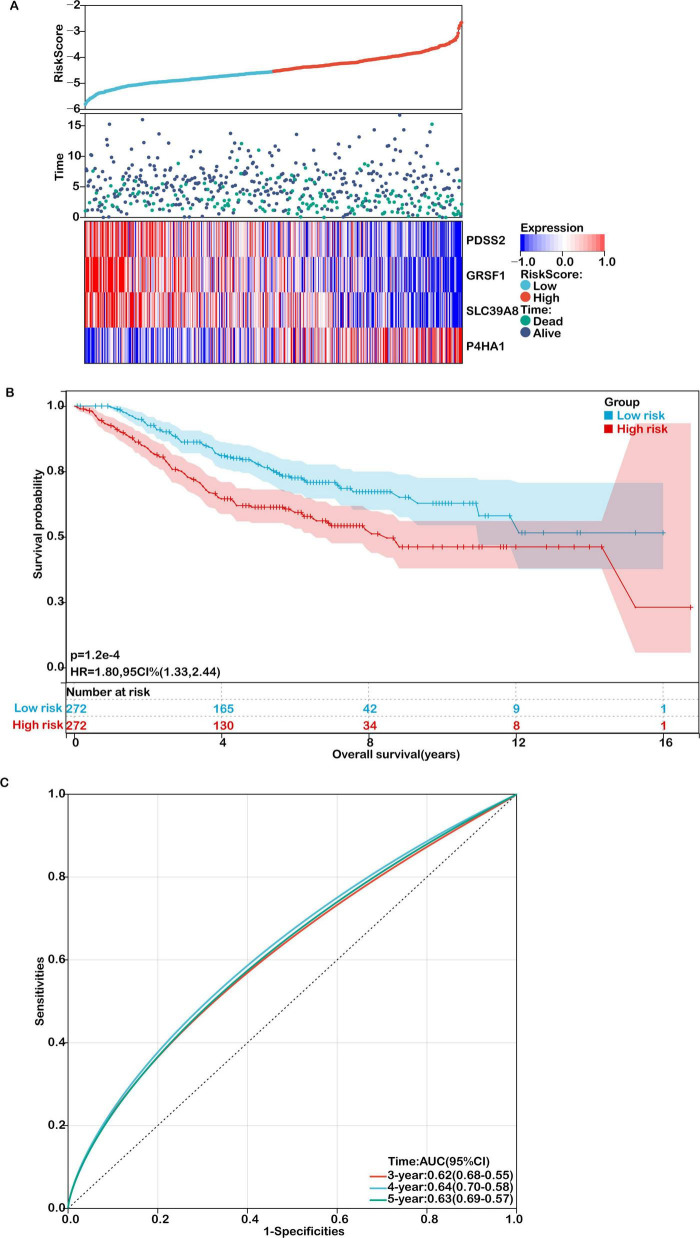
Validation of the prognostic model in an independent cohort. **(A)** Distribution of risk scores, survival status (green for deceased, purple for alive), and gene expression of the 4 model genes in the GSE39582 validation cohort. **(B)** Kaplan-Meier survival curves comparing overall survival between high- and low-risk groups. **(C)** ROC curves for predicting 3-, 4-, and 5-year overall survival in the validation cohort.

The robustness of the model was further confirmed in the external validation cohort GSE39582. As shown in Figures 5a,b, patients with higher risk scores exhibited poorer survival outcomes, consistent with the TCGA results. Expression levels of the four signature genes in this cohort are visualized as heatmaps (Figure 5A), and Kaplan–Meier survival curves again showed significantly reduced OS in high-risk patients (Figure 5B). The AUC values for predicting 3-, 4-, and 5-year survival in the GEO cohort were 0.62, 0.64, and 0.63, respectively (Figure 5C), supporting the model’s generalizability and clinical relevance.

### Development and validation of a prognostic nomogram integrating mitochondrial ferroptosis-associated risk signatures

To facilitate individualized prognosis prediction and enhance clinical decision-making in colon cancer management, we developed a nomogram integrating the mitochondrial ferroptosis-based risk score with key clinical parameters. Both univariate and multivariate Cox regression analyses confirmed that the risk score and pathological TNM (pTNM) stage were independently associated with overall survival (Figure 4C), highlighting their potential as reliable prognostic indicators.

Based on these findings, we constructed a prognostic nomogram that combined the risk score and pTNM stage to estimate 3-, 4-, and 5-year survival probabilities (Figure 4C). Receiver Operating Characteristic (ROC) analysis demonstrated that the nomogram exhibited high predictive performance, with AUC values of 0.82, 0.83, and 0.80 for 3-, 4-, and 5-year survival, respectively (Figure 4D). Calibration plots further verified the agreement between predicted and observed outcomes, supporting the model’s predictive precision. The concordance index (C-index) of the nomogram reached 0.78 (95% CI: 0.73–0.83; *p* < 0.001), indicating strong discriminatory power and clinical applicability (Figure 4E). Comparative analyses were performed to evaluate the model’s predictive performance against established pathological prognostic factors including TNM staging, age and gender (Supplementary Figure S4). The ferroptosis-related gene signature significantly outperformed conventional prognostic markers (TNM staging, age, gender) in predictive accuracy (AUC: 0.82 vs. 0.65/0.61/0.56, respectively; all *p* < 0.01) and demonstrated superior 5-year overall survival stratification (Supplementary Figure S4).

Moreover, we investigated the correlation between the calculated risk scores and clinical staging to assess the model’s stratification capability. As shown in Figure 4F, elevated risk scores were significantly associated with more advanced disease stages. Specifically, patients in stages II–IV exhibited higher risk scores compared to those in stage I, and those in stage III had significantly greater scores than stage II patients (*P* < 0.05). These findings emphasize the prognostic utility of the mitochondrial ferroptosis-related risk signature, particularly in identifying high-risk individuals with advanced colon cancer.

### Association between mitochondrial ferroptosis-related risk score and tumor microenvironment signatures in COAD

To elucidate the molecular differences underlying risk stratification, we performed differential expression analysis between the high- and low-risk patient cohorts. In the high-risk group, genes such as P4HA1, SERPINH1, and BGN were significantly upregulated. These genes are involved in ECM remodeling and the tumor microenvironment (TME). P4HA1 helps stabilize collagen and reorganize the matrix, promoting tumor cell growth and spread. SERPINH1 supports collagen folding and ECM integrity, aiding cancer cell survival. BGN, a proteoglycan, is key for matrix structure and cell signaling, and its abnormal expression is linked to metastasis and immune evasion. These findings highlight the role of ECM pathways in tumor progression and the immunosuppressive environment of high-risk colon cancer.

**FIGURE 6:**
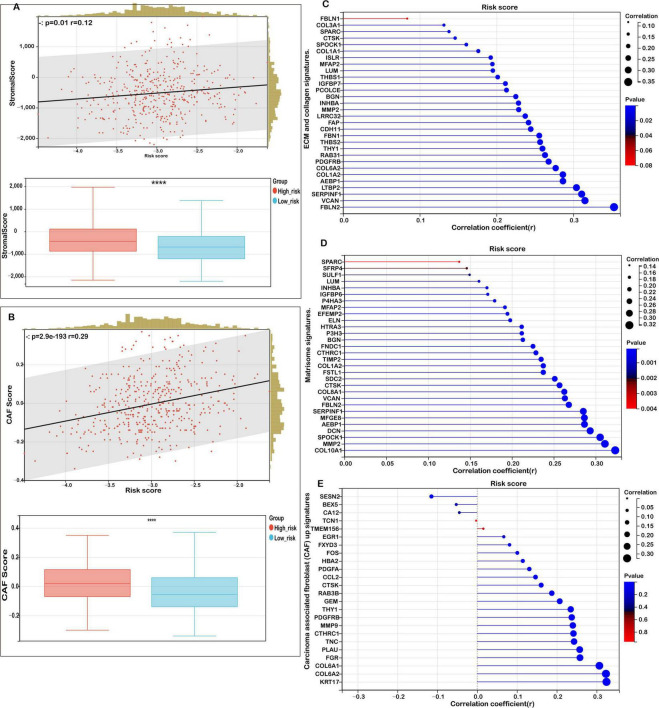
Correlation between risk score and tumor microenvironment (TME) signatures in COAD. **(A)** Correlation analysis between stromal score and risk score, with distribution comparisons between low- and high-risk groups. **(B)** Analysis of the relationship between carcinoma-associated fibroblast (CAF) score and risk score, with distribution comparisons between low- and high-risk groups. **(C)** Correlation of risk scores with extracellular matrix (ECM) and collagen gene signatures. **(D)** Correlation between risk scores and matrisome gene expression. **(E)** Association of the risk score with upregulated and downregulated CAF signatures. Statistical significance is marked as ****P < 0.0001.

Building on these insights, given that TME-associated signaling pathways were enriched in functional analyses, we systematically analyzed the correlation between the risk score and key TME-related signatures. As illustrated in Figure 6A, the mitochondrial ferroptosis-derived risk score demonstrated a strong positive association with stromal scores, with patients in the high-risk group exhibiting significantly elevated stromal content compared to those in the low-risk group.

Further analysis revealed that the risk score was also positively correlated with the abundance of cancer-associated fibroblasts (CAFs), as evidenced by a marked increase in CAF scores among high-risk patients (Figure 6B). This highlights the contributory role of CAFs in shaping the TME and driving colorectal cancer progression.

In addition, we observed robust positive correlations between the risk score and multiple extracellular matrix (ECM)-related gene signatures. Specifically, ECM-collagen signatures (Figure 6C), core matrisome components (Figure 6D), and CAF-associated gene sets (Figure 6E) were all significantly enriched in the high-risk subgroup. These findings suggest that aberrations in mitochondrial ferroptosis may enhance matrix remodeling and fibrosis by modulating the expression of ECM and stromal-associated genes, ultimately fostering a tumor-promoting microenvironment.

In conclusion, our study indicates that elevated mitochondrial ferroptosis-related risk scores are closely linked to increased stromal activation, ECM organization, and CAF infiltration. This suggests a potential mechanism by which mitochondrial dysfunction and ferroptosis dysregulation indirectly facilitate tumor cell proliferation, invasion, and immune evasion through TME modulation.

### The mitochondrial ferroptosis-associated risk score reflects an immunosuppressive tumor microenvironment in high-risk COAD patients

The tumor immune microenvironment (TIME) exerts a profound influence on therapeutic response and clinical prognosis in COAD. To elucidate the immunological landscape associated with mitochondrial ferroptosis-related risk levels, we examined immune cell infiltration patterns in COAD patients using the CIBERSORT algorithm.

**FIGURE 7:**
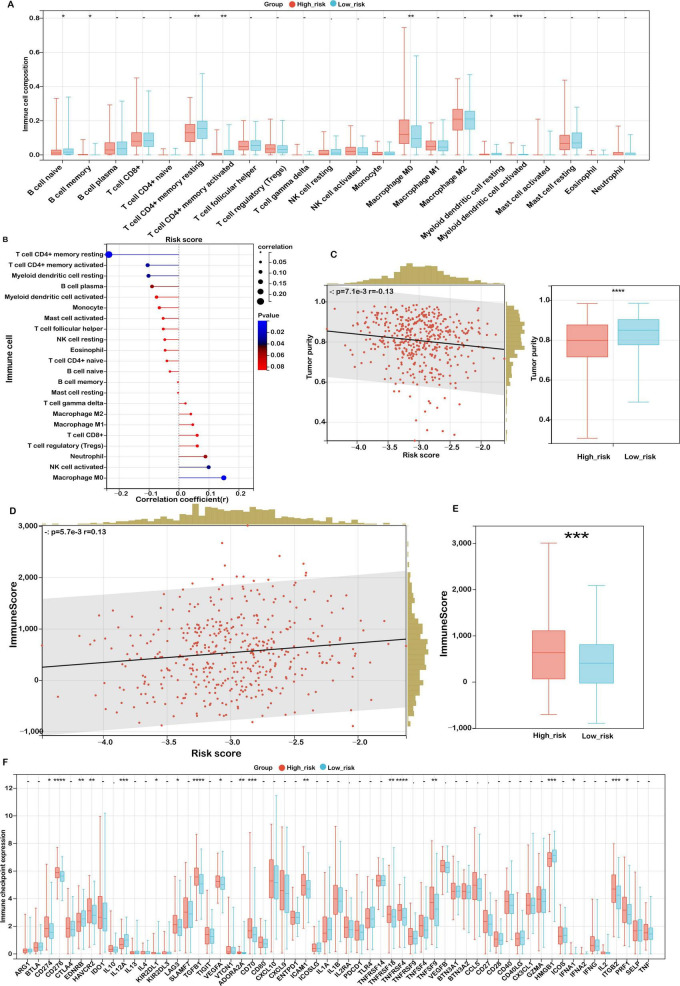
Association between risk score and immune microenvironment characteristics. **(A)** CIBERSORT analysis of immune cell proportions in low- and high-risk groups. **(B)** Correlation between risk score and immune cell infiltration. **(C)** Analysis of the relationship between risk score and tumor purity, with distributions for each risk group. **(D)** Correlation between risk score and immune score. **(E)** Immune scores in low- and high-risk groups. **(F)** Expression variations of immune checkpoints. Significance levels: ****P < 0.0001, ***P < 0.001, **P < 0.01, *P < 0.05, ns (not significant).

As illustrated in Figure 7A, low-risk patients exhibited significantly higher proportions of immune cell subsets such as naïve and memory B cells, resting and activated CD4^+^ memory T cells, as well as both resting and activated myeloid dendritic cells. This immune profile suggests a more active and surveillance-oriented immune microenvironment in the low-risk cohort. Conversely, high-risk patients demonstrated a pronounced increase in M0 macrophages, indicative of a less differentiated and potentially immunosuppressive immune state conducive to tumor progression. These findings further confirm the observed trend in the relationship between risk score and immune cell infiltration (Figure 7B).

To further investigate the characteristics of the tumor microenvironment, the ESTIMATE algorithm was applied to assess tumor purity and stromal/immune content. High-risk patients displayed significantly lower tumor purity (Figure 7C, *P* < 0.001), implying extensive infiltration by stromal and immune cells. Correspondingly, both the stromal score (Figure 7D) and immune score (Figure 7E) were significantly elevated in this group, highlighting an enriched non-tumor cellular milieu.

Building on previous findings, we observed strong positive correlations between the risk score and key signatures of the matrisome and cancer-associated fibroblasts (CAFs), whereas an inverse correlation was observed with activated CD4^+^ T cells. This pattern suggests that a fibroblast-enriched, ECM-dominant microenvironment may hinder T cell activation and facilitate immune evasion in high-risk individuals.

Taken together, these results indicate that high mitochondrial ferroptosis-related risk scores are associated with a complex and immunosuppressive TIME, characterized by stromal overrepresentation, reduced immune activation, and impaired antitumor immunity. These findings underscore the therapeutic potential of strategies targeting stromal components and immune suppression in high-risk COAD patients, warranting further mechanistic and translational investigations.

### Association between mitochondrial ferroptosis-related risk score and immunotherapy response in COAD

Given the growing clinical relevance of immune checkpoint inhibitors (ICIs) in cancer treatment, we investigated the association between mitochondrial ferroptosis-related risk scores and the expression of immune checkpoint molecules in COAD. Our analysis identified 18 immune checkpoint genes that were significantly dysregulated in the high-risk cohort (Figure 7F). Notably, several immune inhibitory molecules, including CD274 (PD-L1), HAVCR2 (TIM-3), LAG3, and TGFB1, were upregulated in the high-risk group. Although not reaching statistical significance, transcriptomic correlation analysis demonstrated a weak yet discernible positive association between SLC39A8—an essential regulator of lipid peroxidation—and PD-L1 (CD274) expression (Supplementary Figure S5; Pearson’s *r* = 0.09, *p* = 0.07). This observation may indicate a potential mechanistic interplay between ferroptosis-related pathways and immune checkpoint regulation. This suggests an immunosuppressive tumor microenvironment that may impede effective antitumor immunity and facilitate immune escape.

Concurrently, elevated levels of co-stimulatory receptors such as TNFRSF4 (OX40) and TNFRSF9 (4-1BB) were also observed, implying a potential, yet insufficient, compensatory immune activation. In contrast, low-risk patients demonstrated increased expression of pro-inflammatory markers like HMGB1 and IL12A, indicating a more immunologically active and anti-tumoral landscape. These observations highlight distinct immune regulatory states across risk subgroups and emphasize the need for personalized immunotherapeutic strategies aligned with individual immune profiles.

**FIGURE 8:**
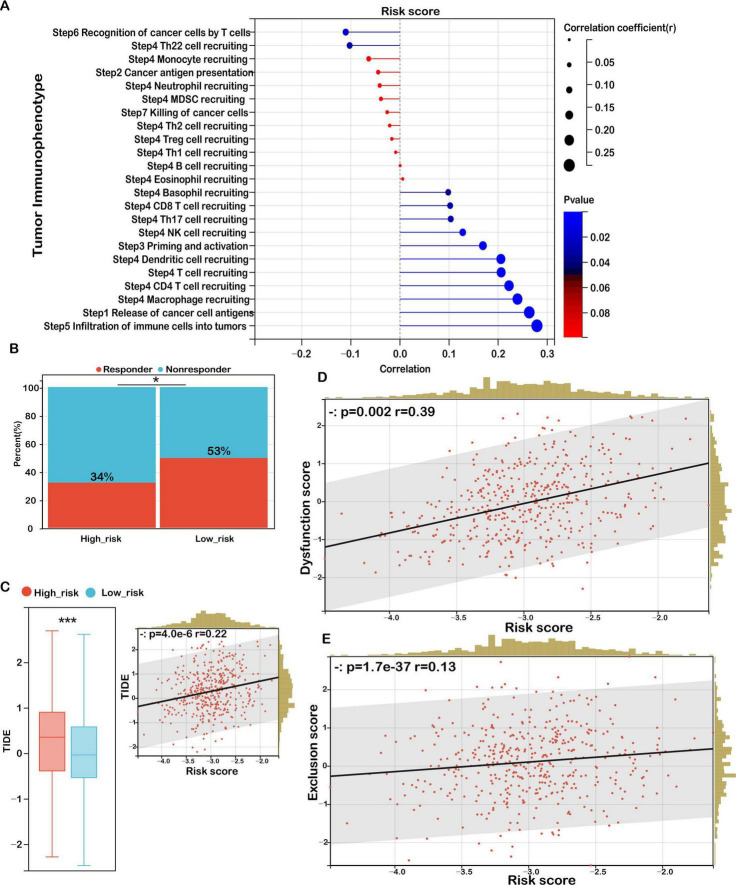
Risk score as a biomarker for predicting immunotherapy benefits in COAD. **(A)** Correlation analysis between risk score and tumor immune phenotype. **(B)** TIDE-predicted immunotherapy response rates for groups stratified by low and high immune scores. **(C)** TIDE scores for low- versus high-risk groups, with their correlation to risk score and association with TIDE scores. **(D)** Correlation between risk score and dysfunction score. **(E)** Correlation between risk score and exclusion score. Significance: ns (not significant); ***P < 0.001, *P < 0.05.

To further dissect the functional immune status, we conducted a tumor immunophenotyping analysis based on the cancer-immunity cycle. As demonstrated in Figure 8A, elevated risk scores were significantly associated with the suppression of essential antitumor immune responses, notably antigen presentation, T cell-mediated recognition of tumor cells (Step 6), and the recruitment of immune effector cells—including Th22 cells, monocytes, neutrophils, myeloid-derived suppressor cells (MDSCs), Th2 cells, regulatory T cells (Tregs), and Th1 cells (Step 4). Moreover, cancer cell killing capacity (Step 7) was also attenuated. In contrast, positive associations were observed in processes such as antigen release, dendritic cell recruitment, CD4 + and CD8 + T cell infiltration, Th17 cell activation, natural killer (NK) cell recruitment, and immune cell priming, reflecting a paradoxical immune response. These patterns suggest that while certain immune mechanisms are activated in high-risk tumors, the impairments in antigen recognition and effector function contribute to immune evasion and tumor progression.

To evaluate potential responses to ICIs, we applied the TIDE (Tumor Immune Dysfunction and Exclusion) framework. The high-risk group exhibited a significantly lower predicted response rate to immunotherapy (34%) compared to the low-risk group (53%) (Figure 8B). Moreover, TIDE scores were significantly higher in high-risk patients and positively correlated with the risk score (Figure 8C), indicating a greater likelihood of immune escape and resistance to checkpoint blockade therapy in this subgroup.

In-depth analysis of the two core TIDE components—T cell dysfunction and T cell exclusion—revealed a strong positive correlation between the risk score and the dysfunction score (*r* = 0.39, *p* = 0.002), suggesting elevated levels of impaired T cells in high-risk tumors (Figure 8D). Additionally, the exclusion score was also positively associated with the risk score (*r* = 0.13, *p* = 1.7 × 10^–37^), indicating an enhanced capacity of these tumors to prevent T cell infiltration into the tumor core (Figure 8E).

These findings corroborate our earlier immune landscape analysis, wherein high-risk tumors were characterized by reduced infiltration of cytotoxic and memory T cells and an increased presence of immunosuppressive elements, such as M0 macrophages and fibroblasts. Collectively, this dual phenotype of immune dysfunction and exclusion may define a highly immune-resistant subtype of colon cancer, potentially limiting the clinical efficacy of immunotherapies in this patient population. This underscores the necessity for combinatorial strategies targeting both the immunosuppressive environment and immune checkpoint pathways to improve treatment outcomes in high-risk COAD patients.

### Mutation landscape of COAD patients stratified by mitochondrial ferroptosis-associated risk score

Somatic mutations are a hallmark of tumorigenesis and play a pivotal role in the initiation and progression of colorectal cancer. With the advent of high-throughput sequencing technologies, it has become possible to comprehensively characterize the mutational spectrum and identify critical oncogenic drivers. In this study, we classified COAD patients into high- and low-risk groups according to their mitochondrial ferroptosis-related gene expression profiles and subsequently analyzed the differences in their mutational landscapes.

**FIGURE 9:**
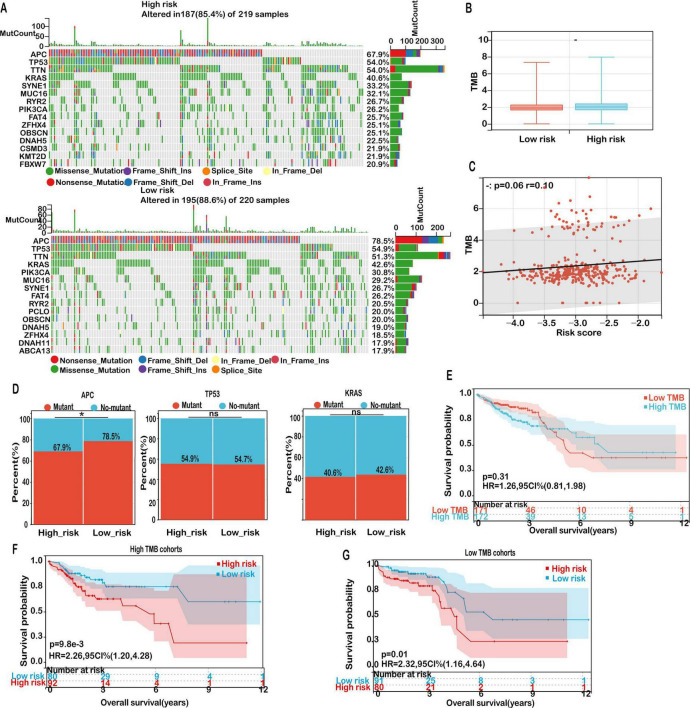
Mutation landscape in high- and low-risk COAD groups. **(A)** Mutation frequencies for the top 15 genes in high- and low-risk groups. **(B)** Distribution of TMB scores in low- versus high-risk groups. **(C)** Correlation analysis between risk score and TMB. **(D)** Mutation rates of the top mutant genes in high-risk and low-risk groups. **(E)** Kaplan-Meier survival curves for patients classified as high TMB and low TMB in the TCGA-COAD cohort. **(F)** Kaplan-Meier survival curves for patients in the high TMB cohort, stratified by TMB score. **(G)** Kaplan-Meier survival curves for patients in the low TMB cohort, stratified by TMB score. Significance: ns (not significant); *P < 0.05.

Our results demonstrated that the 15 most commonly mutated genes—APC, TP53, TTN, KRAS, MUC16, SYNE1, RYR2, FAT4, PIK3CA, and OBSCN—were consistently prevalent in both subgroups (Figure 9A), suggesting that these alterations represent core components of the mutational architecture of COAD, independent of ferroptosis-related risk stratification.

Given the established role of tumor mutation burden (TMB) as a potential biomarker for predicting immunotherapy efficacy, we further evaluated TMB levels across risk groups. Although the high-risk cohort exhibited a modestly elevated TMB compared to the low-risk group, the difference did not reach statistical significance (Figure 9B). Furthermore, correlation analysis revealed a weak but non-significant positive association between risk score and TMB (Figure 9C, *r* = 0.13, *P* = 0.06), indicating that while TMB may be mildly associated with ferroptosis-related risk, it is unlikely to be a dominant driver of immunological divergence in this context.

Notably, subgroup-specific differences were observed in the mutational frequency of APC, a tumor suppressor central to the Wnt/β-catenin signaling pathway. As illustrated in Figure 9D, the mutation frequency of APC was notably higher in the low-risk group (78.5%) than in the high-risk group (67.9%). This observation suggests that alternative oncogenic drivers or immune evasion mechanisms may play a more prominent role in the high-risk cohort. In contrast, mutation frequencies of TP53 and KRAS did not significantly differ between groups, suggesting their ubiquitous involvement across diverse molecular subtypes of COAD.

Despite TMB not showing a significant prognostic association on its own (*P* = 0.31; Figure 9E), further stratified survival analyses provided additional insights. Among patients with high TMB, those in the low-risk group experienced significantly improved overall survival (OS) compared to high-risk counterparts (*P* = 0.0098; Figure 9F). A similar survival benefit was observed within the low TMB subgroup, where low-risk patients also exhibited superior outcomes (*P* = 0.01; Figure 9G). These findings emphasize the prognostic relevance of integrating TMB status with the mitochondrial ferroptosis-associated risk model to refine risk assessment and guide personalized treatment strategies in COAD.

Collectively, our mutation profiling underscores that although the overall mutational spectrum remains largely conserved between high- and low-risk groups, subtle variations—particularly in APC mutation frequency and the interplay with TMB—may underlie the biological heterogeneity and differential prognosis in COAD patients.

### Therapeutic implications of the mitochondrial ferroptosis-associated risk score in COAD

To investigate the potential of the mitochondrial ferroptosis-related gene signature in guiding individualized therapy, we performed a pharmacogenomic analysis aimed at predicting drug responses across different risk groups in colon adenocarcinoma. The analysis revealed distinct drug sensitivity profiles associated with the stratified risk categories, suggesting potential avenues for personalized treatment.

**FIGURE 10:**
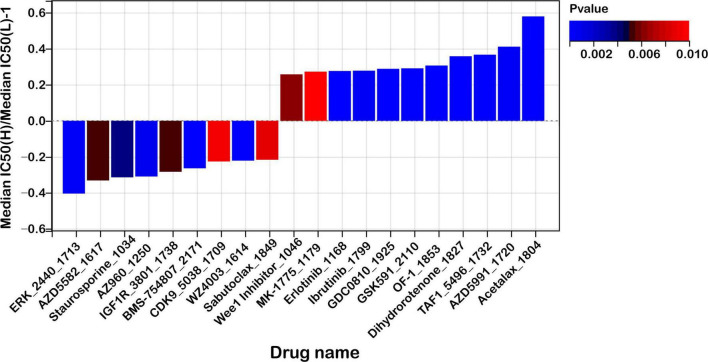
Risk score predicts drug sensitivity in colon cancer. A comparison of normalized IC50 values for the top 10 drugs between high- and low-risk groups, with significant differences observed (*P* < 0.01).

As illustrated in Figure 10, patients classified in the high-risk group exhibited increased susceptibility to compounds such as ERK_2440_1713, AZD5582_1617, and Staurosporine_1034. Among these, AZD5582 acts as a second mitochondrial activator of caspase (SMAC) mimetic that antagonizes inhibitor of apoptosis proteins (IAPs), thereby restoring apoptotic signaling pathways often impaired in aggressive malignancies. Staurosporine, a broad-spectrum kinase inhibitor, is known for its potent pro-apoptotic effects across a wide range of tumor models. Enhanced sensitivity to ERK_2440_1713 in this subgroup points to a potential dependence on aberrant MAPK/ERK pathway activity, which may represent a therapeutically actionable vulnerability.

In contrast, low-risk patients demonstrated greater responsiveness to agents including Acetalax_1804, AZD5991_1720, and TAF1_5496_1732. AZD5991, a selective inhibitor targeting the anti-apoptotic protein MCL-1, promotes mitochondrial-mediated cell death and has shown promising efficacy in preclinical studies. Acetalax modulates members of the BCL-2 family, facilitating intrinsic apoptotic pathways. TAF1_5496_1732 disrupts TAF1, a key transcriptional regulator implicated in cell cycle progression and tumor maintenance, suggesting that transcriptional regulatory mechanisms may be a therapeutic target in low-risk tumors.

Furthermore, we conducted a stratified analysis of the predicted half-maximal inhibitory concentration (IC50) values for commonly used chemotherapeutic agents in colorectal cancer, including 5-fluorouracil (5-FU), irinotecan, and oxaliplatin, between the high- and low-risk groups. However, no statistically significant differences in drug sensitivity were observed between the two groups (Supplementary Figure S6). Further experimental and clinical validation is warranted to clarify potential role of mitochondrial ferroptosis-related genes in guiding therapeutic strategies beyond conventional chemotherapy.

These findings suggest that the ferroptosis-associated risk score may serve as a promising tool within the paradigm of precision oncology by guiding treatment decisions based on predicted drug responsiveness. The observed differences in drug sensitivity profiles between high- and low-risk groups further imply the potential for combinatorial strategies, particularly with immune checkpoint inhibitors, in light of the distinct immune microenvironmental characteristics previously identified.

Integrating the risk score with pharmacological response data may facilitate the development of individualized therapeutic regimens, thereby enhancing treatment efficacy by aligning patients with agents most likely to elicit favorable outcomes based on their molecular and immunological features. However, these associations require further validation through *in vitro* and *in vivo* experiments to confirm their predictive value and support their clinical applicability.

### Correlation between ferroptosis markers and PD-L1 expression

**FIGURE 11:**
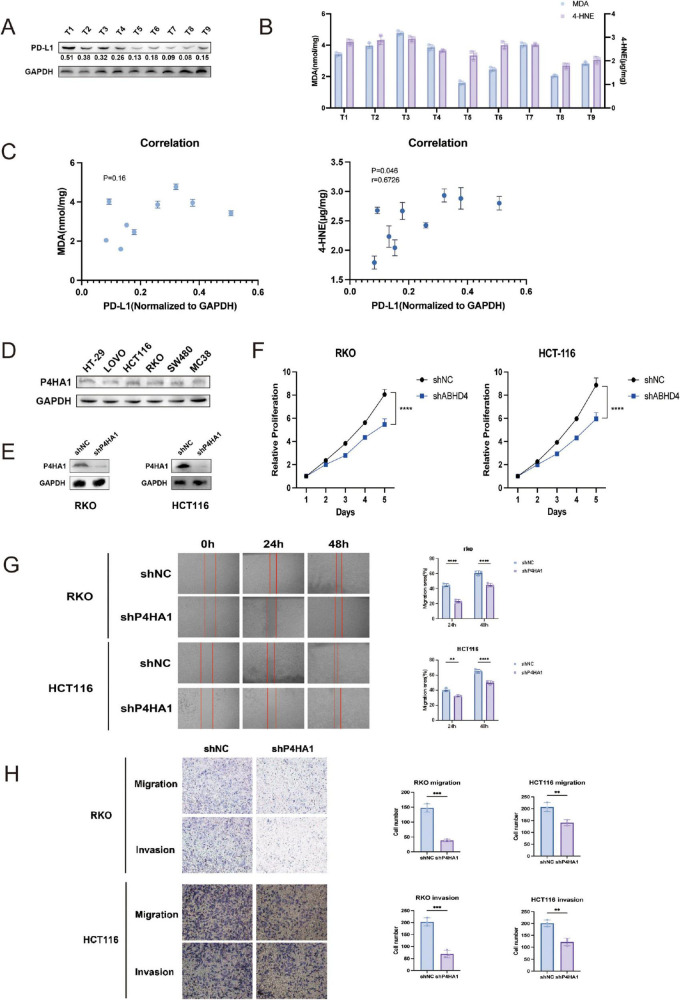
Correlation between ferroptosis markers and PD-L1 expression, and effects of P4HA1 knockdown on CRC cells. **(A)** Western blot analysis of PD-L1 expression in nine paired colorectal cancer (CRC) specimens. **(B)** Quantification of ferroptosis markers malondialdehyde (MDA) and 4-hydroxynonenal (4-HNE) levels in the same CRC samples. **(C)** Spearman correlation analysis showing a positive correlation between PD-L1 and 4-HNE expression (*p* = 0.046), but no significant correlation with MDA (*p* = 0.16). **(D)** Western blot detection of P4HA1 expression across six CRC cell lines. **(E)** Confirmation of P4HA1 knockdown in RKO and HCT116 cells via lentiviral infection. **(F)** Cell viability assays demonstrating significantly reduced proliferation upon P4HA1 knockdown. **(G,H)** Migration and invasion assays indicating decreased motility of CRC cells after P4HA1 silencing. Data are presented as mean ± SD from three independent experiments. Statistical significance is marked as ****P < 0.0001, ***P < 0.001, **P < 0.01.

To explore the relationship between ferroptosis and immune checkpoint regulation, we analyzed nine paired colorectal cancer (CRC) tissue samples for the expression of ferroptosis-related markers malondialdehyde (MDA) and 4-hydroxynonenal (4-HNE), alongside PD-L1 expression. Western blotting was performed to quantify PD-L1 levels (Figure 11A), while the concentrations of MDA and 4-HNE were measured using biochemical assays (Figure 11B). Spearman correlation analysis revealed a significant positive association between PD-L1 expression and 4-HNE levels (*p* = 0.046), whereas the correlation between PD-L1 and MDA was not statistically significant (*p* = 0.16) (Figure 11C). These findings suggest a potential link between ferroptosis-associated lipid peroxidation and PD-L1 expression in CRC.

### P4HA1 knockdown suppresses proliferation and migration of CRC cells *in vitro*

To elucidate the functional significance of P4HA1, a core gene identified in our prognostic signature, we further examined its involvement in colorectal cancer (CRC) progression. Western blot analysis was conducted to evaluate P4HA1 expression across six CRC cell lines (Figure 11D). Subsequently, stable P4HA1 knockdown cell lines (RKO/shP4HA1 and HCT116/shP4HA1) were generated via lentiviral transduction (Figure 11E). Functional assays demonstrated that P4HA1 knockdown significantly reduced CRC cell viability, as shown by decreased proliferation rates (Figure 11F). Moreover, silencing P4HA1 impaired the migratory and invasive capabilities of CRC cells (Figures 11G,H). These results indicate that P4HA1 contributes to CRC cell proliferation and metastasis potential.

## Discussion

Colorectal cancer (CRC) is a major global health concern, ranking as the third most frequently diagnosed malignancy and the second leading cause of cancer-related mortality worldwide (27). The prognosis of CRC is strongly stage-dependent, with a reported 5-year overall survival rate of approximately 65% (28). Ferroptosis, a recently characterized form of regulated cell death, is distinct from apoptosis both morphologically and mechanistically. It is driven by iron accumulation and lipid peroxidation, and was first identified through the use of erastin, a small molecule that induces cell death in tumor cells (29, 30). Unlike apoptosis, ferroptosis is marked by unique ultrastructural mitochondrial changes, such as reduced mitochondrial volume, rupture of the outer membrane, and loss or reduction of cristae, without the chromatin condensation or nuclear fragmentation typically seen in apoptotic pathways (31). The progression of malignancies is frequently accompanied by redox dysregulation, often driven by mutations in oncogenes that govern oxidative stress pathways (31, 32). Cancer cells, owing to their altered iron metabolism, typically exhibit elevated intracellular iron levels, rendering them more susceptible to ferroptotic cell death. Recent evidence suggests that ferroptosis plays a critical role in modulating the proliferation and survival of a wide range of tumors, including diffuse large B-cell lymphoma, clear cell renal cell carcinoma, melanoma, and ovarian cancer (33, 34). Despite these insights, the prognostic implications of mitochondrial ferroptosis-associated genes in CRC remain largely unexplored. To address this knowledge gap, our study focused on identifying ferroptosis-related genes with prognostic potential and establishing a predictive model to aid in early risk assessment and therapeutic decision-making for patients with colon adenocarcinoma (COAD).

While several biomarkers—such as PDSS2, GRSF1, and SLC39A8—have been reported to predict prognosis in CRC (35–37), these markers are often evaluated in isolation, limiting their predictive power. Growing evidence supports the superiority of multi-gene prognostic signatures over single-gene approaches across various cancer types. For example, Wang et al. (38) developed a 13-gene immune-related signature that demonstrated robust prognostic value in CRC (38), while Xu and Pan (19) identified a fibroblast-related gene signature capable of predicting patient outcomes and informing treatment strategies (39). Given the emerging role of ferroptosis dysregulation in tumor development, our study established a novel prognostic risk model based on mitochondrial ferroptosis-associated genes. This model not only effectively stratifies COAD patients by risk but also provides insights into their potential responsiveness to immunotherapy. These findings contribute to a growing body of literature advocating for multi-gene, pathway-specific prognostic tools to enhance precision medicine approaches in colon cancer.

In this study, we systematically compared our prognostic model with several previously published ferroptosis-related signatures, including those reported by Zhu, Nie, and Li et al. (24–26). Unlike earlier models that were primarily constructed based on general ferroptosis-related genes, our model specifically focuses on mitochondrial ferroptosis, a more biologically refined and functionally relevant subset. To the best of our knowledge, this is the first attempt to develop a prognostic model centered on mitochondrial ferroptosis genes in colon cancer.

Our Mitochondrial Ferroptosis-Related Risk Model demonstrated notably improved predictive performance. It achieved an area under the curve (AUC) of 0.74, which is significantly higher than that of the model by Zhu et al. (25) (AUC = 0.582), and also showed clear advantages over the models by Li et al. (24) and Nie et al. (26) (AUC = 0.568). Moreover, we developed a prognostic nomogram integrating the risk score and pTNM stage to estimate survival probabilities (Figure 4C). The nomogram exhibited excellent predictive ability, with AUC values reaching 0.83, further demonstrating the robustness and clinical applicability of our model.

In this study, mitochondrial ferroptosis-associated genes were systematically identified through the integration of gene sets from the Molecular Signatures Database (MSigDB) and transcriptomic and clinical data from The Cancer Genome Atlas (TCGA). A two-step statistical approach was employed, comprising univariate Cox regression to screen for survival-associated genes, followed by least absolute shrinkage and selection operator (LASSO) regression to minimize overfitting. This process yielded a four-gene signature—PDSS2, GRSF1, SLC39A8, and P4HA1—with significant prognostic value in colon adenocarcinoma. These genes were selected not only based on statistical significance but also due to their biological relevance to both mitochondrial function and ferroptosis, as supported by prior literature. Among these genes, P4HA1 exhibited a positive regression coefficient, suggesting its potential role as a risk factor, with higher expression levels correlating with worse clinical outcomes in colorectal cancer. P4HA1 promotes nasopharyngeal carcinoma progression by activating HMGCS1, which inhibits erastin-induced ferroptosis and supports the proliferation and survival of both adherent and ECM-detached tumor cells. This ferroptosis resistance may also contribute to immune evasion by altering the tumor immune microenvironment and impairing immune cell–mediated tumor clearance (40). This finding is consistent with previous studies demonstrating that P4HA1 facilitates tumor progression and metastasis through enhanced collagen synthesis and extracellular matrix remodeling. Notably, inhibition of P4HA1 has been shown to attenuate malignant phenotypes, underscoring its promise as a potential therapeutic target (41). In contrast, PDSS2, GRSF1, and SLC39A8 exhibited negative coefficients, suggesting their protective roles, with higher expression correlating with improved patient survival. Notably, these genes have been previously implicated in tumorigenesis, supporting their candidacy as clinically meaningful biomarkers (42–44). GRSF1 is a mitochondrial RNA-binding protein that maintains mitochondrial function and may influence ferroptosis by regulating mitochondrial gene expression and oxidative metabolism (45). A recent study demonstrated that GRSF1 drives tumorigenesis and EMT-mediated metastasis in gastric cancer by activating the PI3K/AKT pathway, and may also facilitate immune evasion through modulation of the tumor immune microenvironment (46). PDSS2 encodes a mitochondrial enzyme critical for CoQ10 biosynthesis, which modulates oxidative stress and ferroptotic sensitivity (47). In immune suppression aspect, enhancing coenzyme Q synthesis—by upregulating PDSS2—was found to boost mitochondrial respiration, facilitate the development of memory T cells after viral infection, and strengthen antitumor immune responses (42). SLC39A8 functions as a metal ion transporter affecting intracellular iron and manganese levels, influencing ferroptotic susceptibility. Elevated SLC39A8 expression enhances lipid peroxidation while downregulating GPX4, FTH1, indicating that SLC39A8-driven ferroptosis plays a crucial role in the depletion of monocytes during sepsis (48). These biological roles underscore the plausibility of their contribution to mitochondrial ferroptosis and COAD progression.

Additionally, the knockdown of P4HA1 in CRC cell lines resulted in marked suppression of cell proliferation, migration, and invasion, underscoring its potential role in tumor progression. Given the scarce reports on P4HA1 function in CRC, our data provide novel insights into its contribution to cancer pathogenesis. These results support further investigation into P4HA1 as a potential therapeutic target and warrant exploration of the mechanistic links between ferroptosis and immune evasion in colorectal cancer.

To assess the model’s predictive performance, both internal and external validations were conducted. ROC analysis showed good diagnostic accuracy (AUC = 0.75), and Kaplan–Meier analysis confirmed that high-risk patients had significantly worse overall survival. External validation using GEO datasets yielded consistent results, supporting the model’s robustness and generalizability in COAD prognosis.

DEG analysis between risk groups showed significant enrichment in extracellular matrix (ECM)-related processes. Notably, P4HA1 contributes to collagen stabilization and matrix remodeling, thereby promoting tumor progression and metastasis (49). SERPINH1 enhances ECM integrity by supporting proper collagen folding, facilitating tumor cell survival (50). BGN, a key regulator of ECM architecture and signaling, has been implicated in metastatic dissemination and immune evasion when aberrantly expressed (51). This aligns with previous literature highlighting the role of ECM accumulation in tumor aggressiveness and its correlation with unfavorable prognosis across various cancer types (52–55). The tumor microenvironment (TME), shaped by the intricate interplay between immune cells and stromal elements such as fibroblasts, is a critical determinant in CRC progression (56, 57). Within this context, normal fibroblasts are reprogrammed into cancer-associated fibroblasts (CAFs), which are prevalent in both primary and metastatic colorectal lesions. These CAFs exhibit high adaptability and actively participate in tumor progression by remodeling the ECM and modulating immune infiltration (58–60).

The matrisome, which encompasses genes encoding core ECM proteins and structural regulators, has emerged as a key element in cancer biology (61). For instance, Yuzhalin et al. (62) developed a nine-gene ECM-based signature that demonstrated prognostic potential across diverse malignancies, further underscoring the clinical relevance of ECM-related genes (62). Consistently, our data revealed a strong positive correlation between the derived risk score and the expression of genes related to CAFs, ECM remodeling, and the matrisome. Moreover, risk score was positively associated with stromal score and inversely related to tumor purity, implying enhanced stromal cell infiltration in the TME of high-risk patients. These observations further corroborate previous findings that stromal components play pivotal roles in determining colon cancer prognosis.

Immune infiltration within the TME also exerts a substantial influence on tumor progression and responsiveness to therapy. A growing body of evidence suggests that specific TME immune phenotypes are associated with differential responses to immunotherapy and divergent clinical outcomes (63–65). One major advantage of immunotherapeutic interventions is their capacity to activate memory CD8^+^ T cells, which mediate long-term antitumor immunity and reduce recurrence and metastasis (66–69). Furthermore, recent studies have reported that TME immune composition can significantly affect survival rates and immunotherapeutic efficacy (70, 71). In light of these findings, we further explored the immune landscape between high- and low-risk groups, aiming to elucidate the differential immune cell distributions and their potential implications for treatment responsiveness.

In this study, we applied the CIBERSORT algorithm, a deconvolution-based technique that estimates the relative abundance of various immune cell subsets from gene expression data, to analyze the immune profile of different risk groups. Our analysis revealed distinct immune cell distribution patterns between high- and low-risk groups, suggesting the presence of unique immunological mechanisms that may contribute to tumor progression.

The tumor immune microenvironment (TIME) plays a crucial role in shaping both therapeutic outcomes and prognosis in COAD. By examining immune cell infiltration using CIBERSORT, our study highlighted significant differences in immune landscapes between the high-risk and low-risk categories of COAD. The low-risk group exhibited an enriched presence of B cell subsets, CD4 + memory T cells, and myeloid dendritic cells, reflecting a more active and vigilant immune context, which may enhance prognosis and therapeutic response, particularly to immunotherapy. In contrast, the high-risk group demonstrated a dominance of M0 macrophages, a marker of immunosuppressive or pro-tumoral immune phenotypes. This macrophage profile could promote tumor progression and contribute to resistance against treatment. These findings underscore the importance of incorporating immune profiling into risk assessment and therapeutic decision-making for COAD patients.

In the present study, immune cell infiltration and immune evasion were assessed using CIBERSORT and TIDE algorithms based on bulk RNA-seq data. These methods are widely adopted and validated for large-scale transcriptomic analyses. However, it is acknowledged that bulk RNA-based approaches are limited by their lack of single-cell resolution and their inability to fully capture the spatial and functional heterogeneity of immune cells within the tumor microenvironment. Advanced techniques such as single-cell RNA sequencing and multiplex immunohistochemistry can provide more refined and dynamic insights into the immune landscape. Incorporating such technologies in future studies may further elucidate the complex immune mechanisms underlying colorectal cancer progression and therapeutic response.

Our analysis of tumor immunophenotypes, grounded in the cancer immunity cycle, revealed a complex relationship between immune processes and the calculated risk score. High-risk patients exhibited marked negative associations with critical antitumor immune processes, including antigen presentation, T cell-mediated tumor recognition (Step 6), and the recruitment of immune effector populations such as Th22 cells, monocytes, neutrophils, myeloid-derived suppressor cells (MDSCs), Th2, Treg, and Th1 cells (Step 4). Additionally, these patients demonstrated impaired capacity for effective tumor cell elimination (Step 7). These results suggest that high-risk patients may experience a functional deficit in these critical immune steps, which can impair anti-tumor immunity. In contrast, positive correlations were observed for the recruitment and activation of effector immune cells such as CD8 T cells, Th17 cells, and natural killer (NK) cells, along with enhanced dendritic cell priming, CD4 T cell activation, and macrophage recruitment, all of which are key to effective immune responses. This dual pattern indicates that while some immune processes are activated in high-risk patient, the overall dysregulation—especially in antigen presentation and T cell-mediated tumor recognition—likely contributes to an immunosuppressive environment that favors tumor progression and immune evasion.

Monoclonal antibodies targeting immune checkpoint molecules have revolutionized cancer therapy, offering significant therapeutic potential (72). Our analysis of immune checkpoint molecules revealed distinct differences between the high- and low-risk colorectal cancer groups, which may explain their divergent immune profiles. In the high-risk group, we observed a substantial upregulation of several immune-suppressive and regulatory molecules, including PD-L1 (CD274), CD276, TIM-3 (HAVCR2), LAG3, and TGFB1, all of which are associated with an immunosuppressive tumor microenvironment. This environment is conducive to T cell exhaustion and immune evasion. Although not statistically significant, transcriptomic correlation analysis revealed a weak positive association between SLC39A8, a key regulator of lipid peroxidation, and PD-L1 (CD274) expression (Supplementary Figure S5, Pearson’s *r* = 0.09, *p* = 0.07), suggesting a potential link between ferroptosis-related pathways and immune checkpoint regulation. Additionally, the co-upregulation of other immune-related genes such as VEGFA, PRF1, and TNFRSF family members further suggests a complex immunoregulatory network that could reduce the effectiveness of immune checkpoint blockade therapies. In contrast, the low-risk group demonstrated elevated HMGB1 expression, which may indicate enhanced innate immune activation. These findings emphasize the potential of risk stratification to guide personalized immunotherapeutic strategies for colon cancer patients. Given the observed differences in immune checkpoint molecule expression between high- and low-risk colorectal cancer groups, future studies are warranted to elucidate the underlying mechanisms driving these divergent immune landscape.

TIDE (Tumor Immune Dysfunction and Exclusion) scoring serves as a crucial predictor of immunotherapy responsiveness, with higher scores correlating to reduced response rates. In our study, we found that an increase in the risk score was associated with a significant decrease in immunotherapy response rates. Specifically, the low-risk group exhibited a response rate of 53%, while the high-risk group showed only a 34% response rate, further confirming the predictive value of the risk score. TIDE analysis also provided deeper insights into the immune evasion mechanisms of high-risk colorectal tumors. The positive correlation between the risk score and both T cell dysfunction and exclusion scores suggests that high-risk tumors employ a dual mechanism of immune resistance. This, coupled with reduced effector T cell infiltration and an increased presence of immunosuppressive cell populations, indicates that high-risk tumors harbor a microenvironment that actively impedes immune surveillance and likely demonstrates poor responses to immunotherapies. These results highlight the potential clinical utility of the fibroblast-based risk model not only for prognosis but also for guiding immunotherapeutic decisions in colon cancer treatment.

Our findings indicate a notable association between ferroptosis and immune regulation in CRC, as evidenced by the positive correlation between PD-L1 expression and 4-HNE levels. This suggests that ferroptosis-related lipid peroxidation may influence the tumor immune microenvironment, potentially impacting immune checkpoint expression. However, the lack of significant correlation with MDA highlights the complexity of ferroptosis pathways and their varied molecular markers.

Our analysis of genetic mutations revealed that although the overall genetic profile of high- and low-risk COAD patients remains largely similar, distinct variations—such as a higher frequency of APC mutations in the low-risk group—may reflect underlying biological differences. While the tumor mutation burden (TMB) demonstrated a weak positive correlation with the risk score, the relationship was not statistically significant, indicating that TMB alone might not capture the full complexity of immune or prognostic factors in these patients. Nevertheless, survival analyses based on stratified TMB data and the mitochondrial ferroptosis-related risk score revealed that combining both markers enhances prognostic accuracy. These findings support the idea that, although major driver mutations are commonly shared across both risk groups, subtle variations in TMB and specific gene mutations like APC may contribute to differences in tumor behavior and immune microenvironments. Furthermore, the mitochondrial ferroptosis-related risk score may offer valuable insights for guiding treatment decisions in COAD patients.

Beyond its prognostic and immunological relevance, the mitochondrial ferroptosis-related risk signature shows promising potential for personalized therapy guidance. Pharmacogenomic analysis indicated that high-risk tumors may be more responsive to treatments targeting apoptotic resistance and the ERK signaling pathway, such as SMAC mimetics and kinase inhibitors. Conversely, low-risk tumors exhibited heightened sensitivity to agents that target anti-apoptotic and transcriptional regulation, including BCL-2/MCL-1 inhibitors and TAF1 inhibitors. These varying drug sensitivities highlight the biological diversity within the risk groups and underscore the potential for using the risk score to guide therapeutic interventions. Further preclinical studies are required to validate these findings and explore their clinical applicability.

### Limitations and future directions

Despite the comprehensive bioinformatics analyses performed in this study, several limitations should be acknowledged. First, the risk model and associated findings were derived from retrospective transcriptomic datasets (TCGA and GEO), which may introduce inherent biases. Prospective validation in larger, independent clinical cohorts is necessary to confirm the robustness and generalizability of the model. Second, although we identified key mitochondrial ferroptosis-related genes (PDSS2, GRSF1, SLC39A8, and P4HA1) and analyzed their potential roles in immune modulation, the mechanistic links between these genes and ferroptosis-mediated immune suppression remain largely inferred from correlative analyses and pathway enrichment. Moreover, our analysis is based on bulk transcriptomic data, which does not capture intratumoral heterogeneity or spatial context—important factors in colorectal cancer progression. Incorporating spatial transcriptomics and single-cell technologies will be valuable for future research to refine the model’s clinical relevance. Functional validation through *in vitro* and *in vivo* experiments is required to elucidate the underlying biological mechanisms. Third, our drug sensitivity predictions were based on *in silico* IC50 estimations and lacked validation using patient-derived or experimental pharmacological data, limiting the direct clinical applicability of these findings.

Future studies should aim to incorporate spatial transcriptomics, proteomics, and multiplex immunofluorescence to clarify the spatial and temporal interactions between ferroptosis markers and immune regulatory pathways, such as PD-L1. Additionally, integrating single-cell sequencing and patient-derived organoids may enhance the understanding of tumor heterogeneity and refine personalized therapeutic strategies guided by the risk model.

## Conclusion

In this study, we established and validated a prognostic risk model utilizing mitochondrial ferroptosis-related genes, which successfully predicts overall survival in patients with COAD. The model shows significant correlations with immune cell infiltration, immune escape mechanisms, and immune checkpoint expression. It also has the potential to forecast responses to both chemotherapy and immunotherapy. Additionally, we observed distinct variations in mutational landscapes and drug sensitivities between the high- and low-risk groups, providing new perspectives for personalized treatment strategies in COAD.

## Data Availability

The datasets presented in this study can be found in online repositories. The names of the repository/repositories and accession number(s) can be found in the article/Supplementary material.

## Footnotes

1
http://www.zhounan.org/ferrdb

2
https://www.broadinstitute.org/mitocarta/mitocarta30-inventory-mammalian-3mitochondrial-proteins-and-pathways

3
http://www.gsea-msigdb.org
