# Supplementary material for: A mitochondrial ferroptosis-related gene signature predicts prognosis and immune landscape in colon cancer
Source: Front Med (Lausanne). 2025 Sep 1;12:1614012. doi: 10.3389/fmed.2025.1614012 (PMC12434033; doi:10.3389/fmed.2025.1614012)
Supplement: Supplementary file 1 [file Data_Sheet_1.pdf]

A Mitochondrial Ferroptosis-Related Gene Signature Predicts Prognosis and Immune Landscape in Colon Cancer

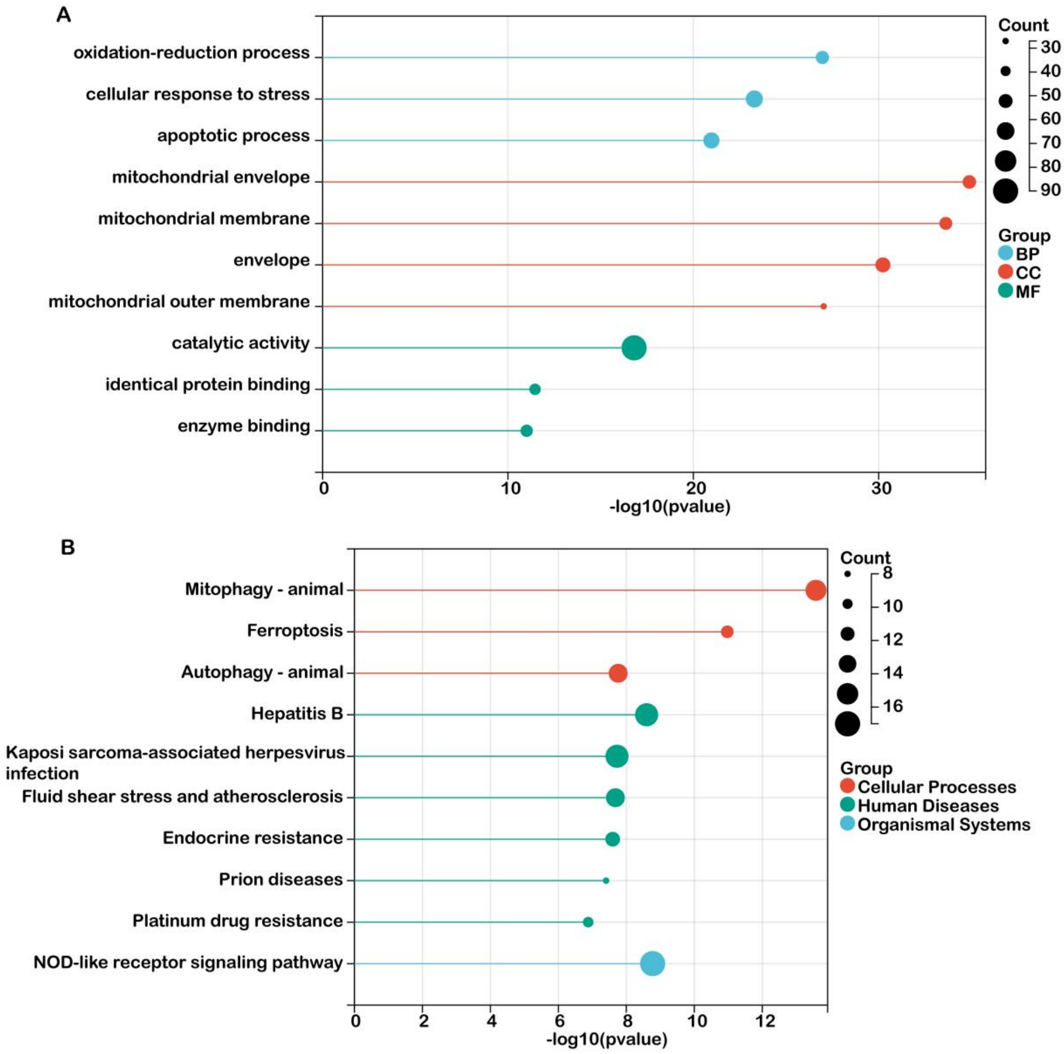

Figure S1. Identification of DEGs related to mitochondrion ferroptosis and functional enrichment analysis in COAD.

(A) The GO analysis of 151 mitochondrial-related DEGs, including biological process (BP), cellular component (CC), and molecular function(MF). **(B)** The KEGG analysis of 220 mitochondrial-related DEGs.

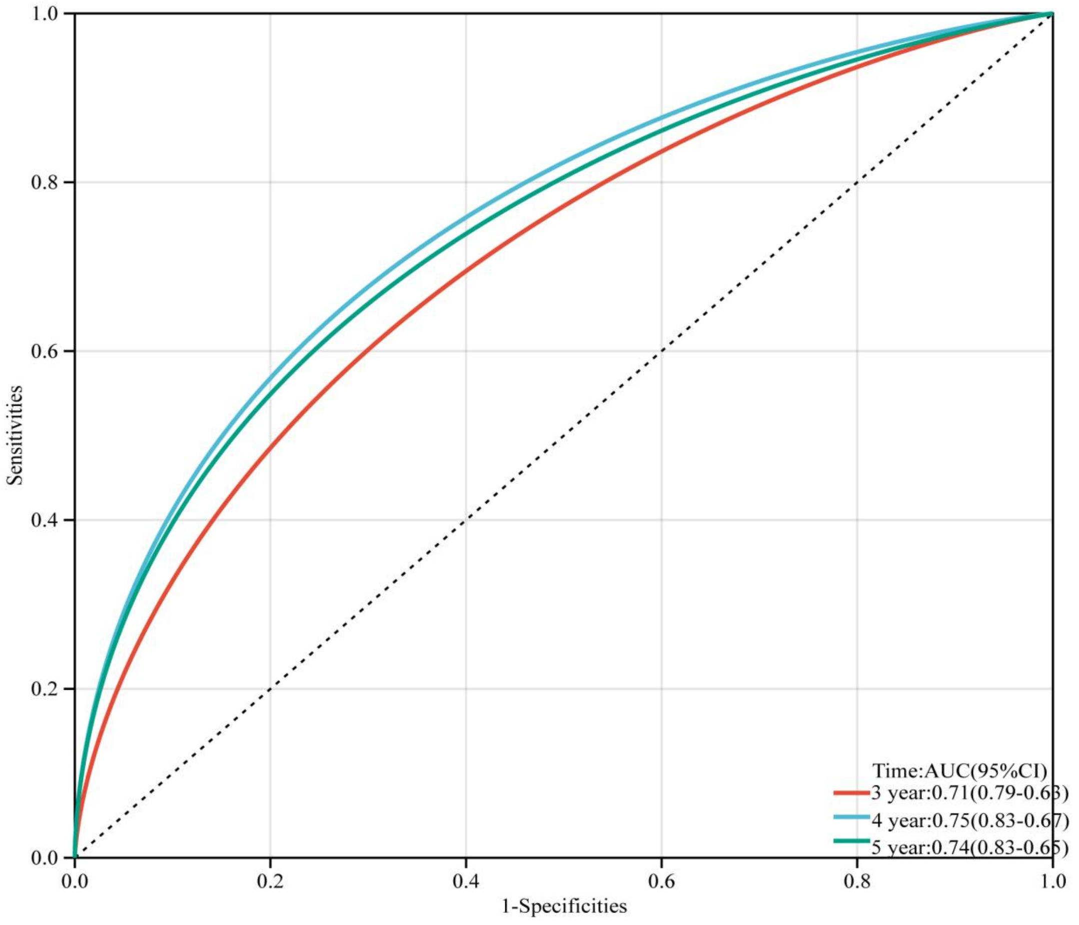

Figure S2. ROC curves for predicting 3-, 4-, and 5-year overall survival in the TCGA cohort.

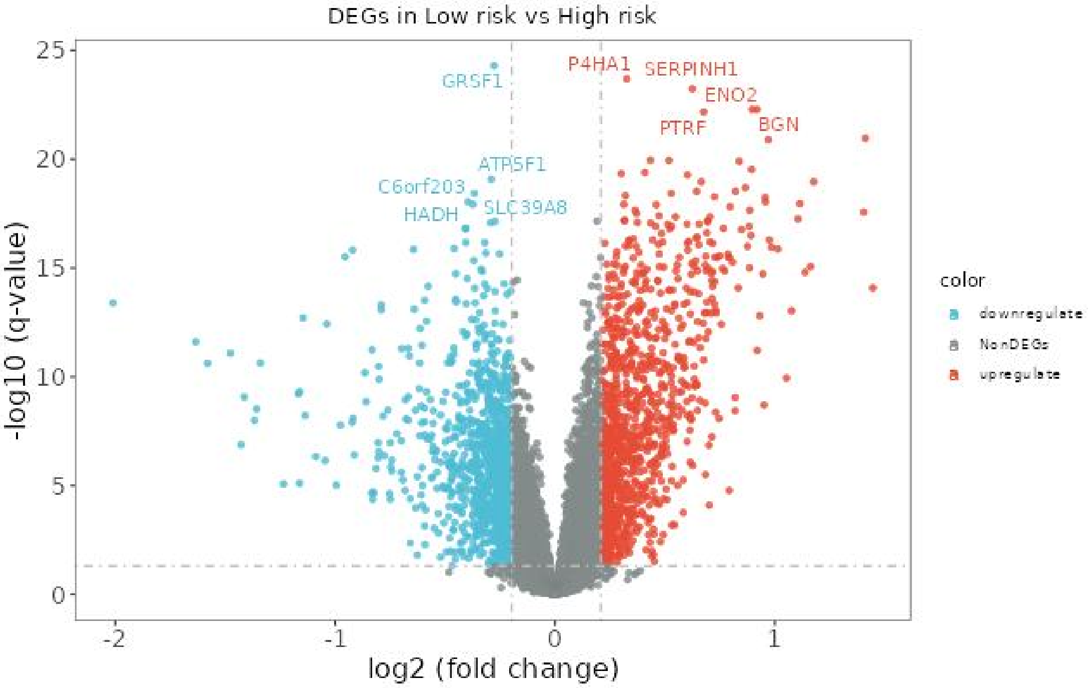

Figure S3. Differential Gene Expression (DEGs) in high-risk and low-risk groups

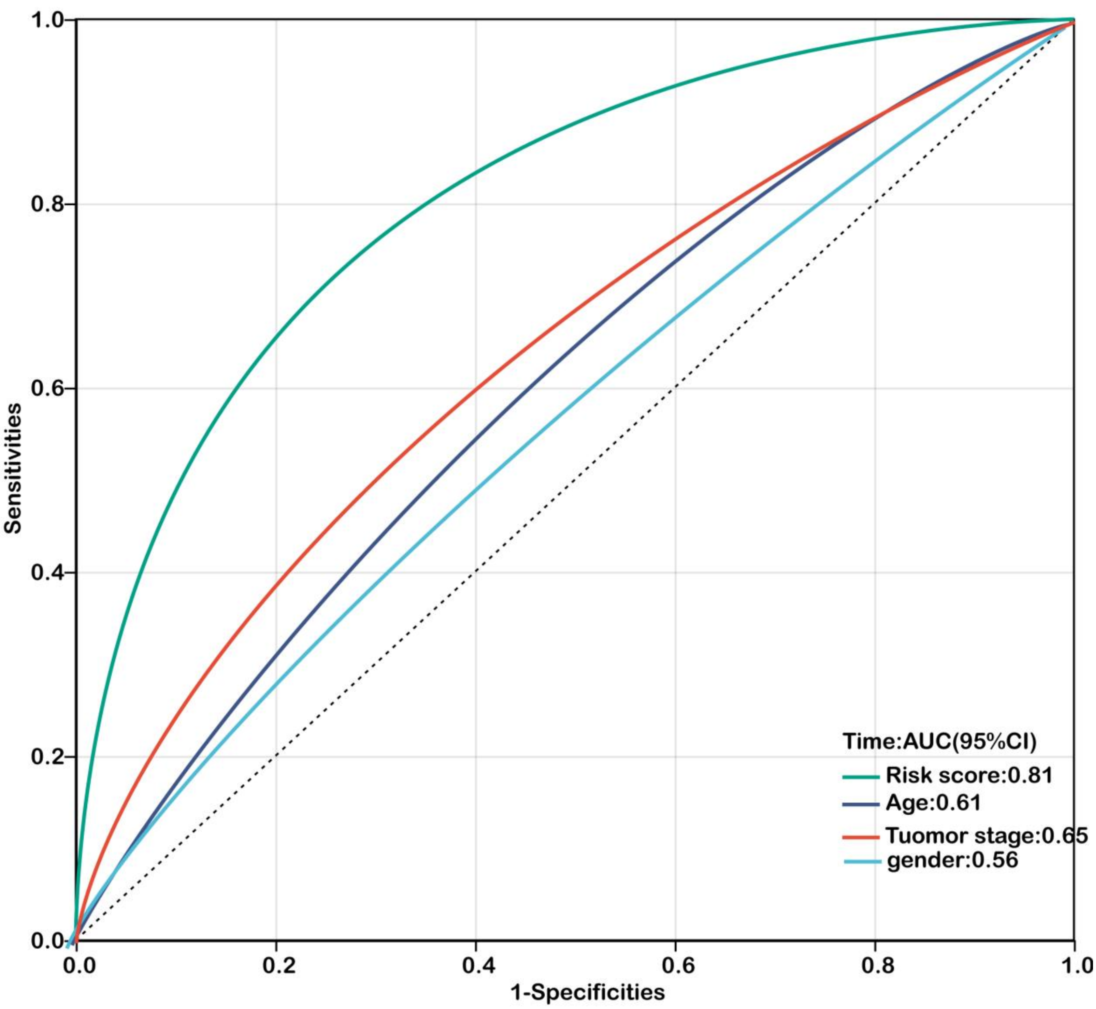

Figure S4. ROC analysis demonstrating superior prognostic performance of the ferroptosis-related risk model over conventional clinicopathological parameters in COAD

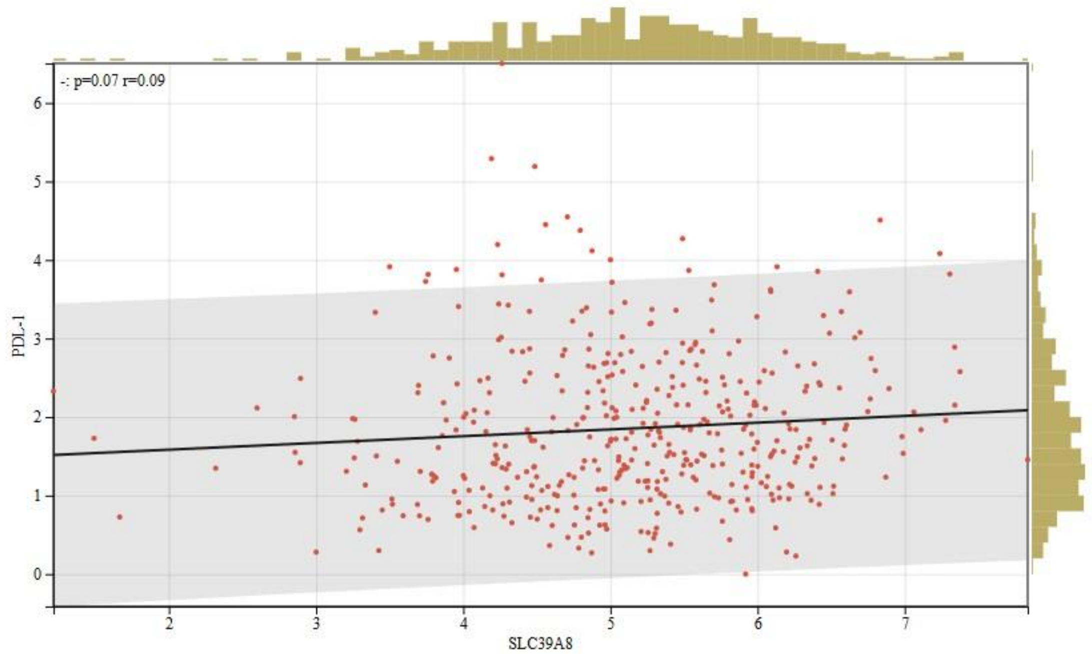

Figure S5.A transcriptomic correlation analysis identified a weak positive association between SLC39A8 and PD-L1 expression

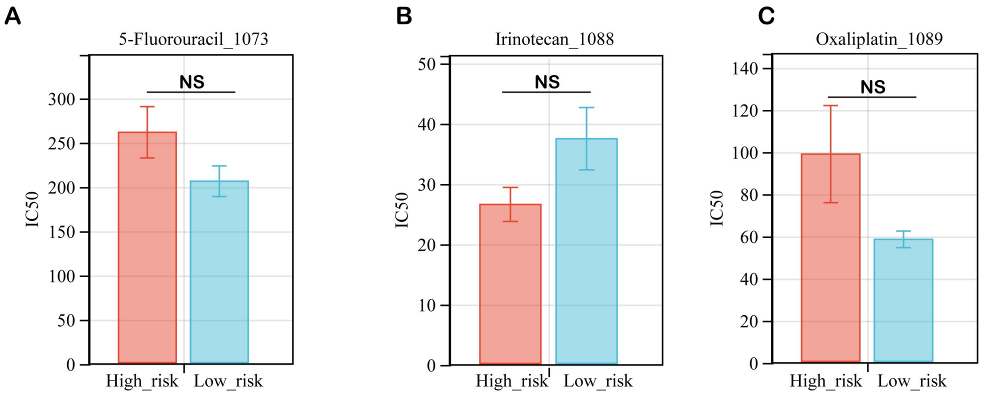

Figure S6.Comparative analysis of the sensitivity to standard chemotherapeutic agents, including 5-fluorouracil (5-FU), irinotecan, and oxaliplatin, between high- and low-risk groups.
